# Recognition memory in amnestic-mild cognitive impairment: insights from event-related potentials

**DOI:** 10.3389/fnagi.2013.00089

**Published:** 2013-12-11

**Authors:** David A. Wolk, Katharine Manning, Daria Kliot, Steven E. Arnold

**Affiliations:** ^1^Department of Neurology, University of Pennsylvania, PhiladelphiaPA, USA; ^2^Penn Memory Center, University of Pennsylvania, PhiladelphiaPA, USA; ^3^Department of Psychiatry, University of Pennsylvania, PhiladelphiaPA, USA

**Keywords:** memory, recollection, familiarity, event-related potentials, FN400, LPC, mild cognitive impairment, Alzheimer’s disease

## Abstract

Episodic memory loss is the hallmark cognitive dysfunction associated with Alzheimer’s disease (AD). Amnestic mild cognitive impairment (a-MCI) frequently represents a transitional stage between normal aging and early AD. A better understanding of the qualitative features of memory loss in a-MCI may have important implications for predicting those most likely to harbor AD-related pathology and for disease monitoring. Dual process models of memory argue that recognition memory is subserved by the dissociable processes of recollection and familiarity. Work studying recognition memory in a-MCI from this perspective has been controversial, particularly with regard to the integrity of familiarity. Event-related potentials (ERPs) offer an alternative means for assessing these functions without the associated assumptions of behavioral estimation methods. ERPs were recorded while a-MCI patients and cognitively normal (CN) age-matched adults performed a recognition memory task. When retrieval success was measured (hits versus correct rejections) in which performance was matched by group, a-MCI patients displayed similar neural correlates to that of the CN group, including modulation of the FN400 and the late positive complex (LPC) which are thought to index familiarity and recollection, respectively. Alternatively, when the integrity of these components was measured based on retrieval attempts (studied versus unstudied items), a-MCI patients displayed a reduced FN400 and LPC. Furthermore, modulation of the FN400 correlated with a behavioral estimate of familiarity and the LPC with a behavioral estimate of recollection obtained in a separate experiment in the same individuals, consistent with the proposed mappings of these indices. These results support a global decline of recognition memory in a-MCI, which suggests that the memory loss of prodromal AD may be qualitatively distinct from normal aging.

## INTRODUCTION

Qualitative aspects and neural correlates of memory impairment in early Alzheimer’s disease (AD) remain to be fully elucidated. Such characterization may have implications for accurate early diagnosis, disease monitoring, and potential therapeutic strategies. Amnestic mild cognitive impairment (a-MCI) is conceptualized as a transitional state between normal aging and the development of clinical AD (Petersen, 2004; Winblad et al., 2004). While a somewhat heterogeneous population, a-MCI is enriched in patients with AD pathology and high likelihood of developing clinical AD (Petersen et al., 2009).

Work that has investigated memory impairment in this population has previously been framed from the perspective of the dual process model (Mandler, 1980; Jacoby, 1991; Yonelinas, 2002). These models generally argue that recognition memory is subserved by the dissociable processes of familiarity and recollection. Familiarity is conceptualized as being a strength-based process, best described by signal detection theory, and phenomenologically associated with an acontextual sense of prior encounter. Recollection is a more lucid, associative form of memory that involves retrieval of contextual aspects of a prior event (e.g., when or where the event occurred). While descriptive details differ across dual process proposals and controversy remains regarding these models (Squire et al., 2007; Wixted et al., 2010), it has served as a useful framework to test memory function in impaired populations, which, in turn, provide additional insight into the validity of this conceptualization.

Somewhat inconsistent findings have been reported for the relative impairment of familiarity and recollection in a-MCI (Westerberg et al., 2006; Anderson et al., 2008; Wolk et al., 2008, 2013; Algarabel et al., 2009, 2012; Ally et al., 2009a; Serra et al., 2010). While almost all work suggests that recollection is significantly impaired in this population, studies have reported a range of effects on familiarity, from complete sparing (Westerberg et al., 2006; Anderson et al., 2008; Serra et al., 2010) to a level of impairment similar to that of recollection (Wolk et al., 2008, 2013). The relative effect of early AD on these memory processes has considerable implications for dual process models that have specified anatomical substrates within the medial temporal lobe (MTL) that dissociably support these memory states (Aggleton and Brown, 2006; Eichenbaum et al., 2007; Ranganath, 2010). The hippocampal formation has generally been argued to be critical to the contextual memory of recollection, and its impairment in early AD is consistent with the relatively early pathological involvement in this structure. Alternatively, familiarity has been argued to be dependent on extrahippocampal MTL structures, particularly perirhinal cortex (Aggleton and Brown, 2006; Eichenbaum et al., 2007; Ranganath, 2010). This region is the earliest associated with the neurofibrillary pathology (neurofibrillary tangles, NFTs) of AD (Braak and Braak, 1991; Delacourte et al., 1999). Thus, the integrity of familiarity-based memory in this population provides important data to evaluate these anatomic mappings.

Event-related potentials (ERPs) have provided some of the strongest support for the dual process model, by providing evidence of a temporal dissociation of the neural correlates of recollection and familiarity (for review, see Rugg and Curran, 2007). In general, ERPs are more positive for correctly recognized items (“hits”) on a memory task than for correct responses to novel items (“correct rejections”) beginning approximately 300 ms after stimulus onset. An early component of this “old/new” effect, often referred to as the “early old/new effect” or FN400, has been associated with familiarity-based responses. For example, items introspectively endorsed as “old,” but without contextual retrieval, are associated with modulation of this component (Duzel et al., 1997; Curran, 2004; Azimian-Faridani and Wilding, 2006; Wolk et al., 2006; Woodruff et al., 2006). This effect tends to occur between 300 and 500 ms with a fronto-central scalp distribution. A later component, sometimes referred to as the “parietal old/new effect,” or late positive complex (LPC), occurs between 500 and 800 ms and is associated with contextual or associative retrieval, consistent with recollection (Wilding and Rugg, 1996; Duzel et al., 1997; Curran et al., 2001; Vilberg et al., 2006; Woodruff et al., 2006; Wolk et al., 2006). This old/new effect tends to have a predominance over left parietal scalp sites. Finally, a number of studies have described a slow wave, sometimes referred to as the late frontal effect (LFE), which occurs at approximately 800 ms, lasts up to 1000 ms, and is often maximal at right hemisphere scalp sites. Modulation of this effect has been argued to be associated with retrieval monitoring or other aspects of executive control during memory retrieval (Wilding and Rugg, 1997; Allan et al., 2000; Ranganath and Paller, 2000; Curran et al., 2001; Ally et al., 2008). An enhanced LFE has previously been associated with compensatory neural activity in older adults and patients with MCI (Ally et al., 2009b; Wolk et al., 2009).

Surprisingly, very few studies have examined recognition memory in a-MCI with ERPs and those have reported somewhat mixed results regarding the integrity of the above old/new effects (Olichney et al., 2008; Ally et al., 2009b; Schefter et al., 2012; Hoppstadter et al., 2013). The current experiment measured ERPs of recognition memory during a task in which participants were first asked to decide whether a word was previously studied and then to determine the color font at study for those items endorsed as “old.” The latter was instituted to encourage retrieval of both familiarity *and* recollection. In this context, we addressed the following issues:

1. Does the neural signature of accurate memory performance, that is “retrieval success” (operationalized as “hits” versus “correct rejections”), differ between a-MCI and healthy older controls? In particular, do these groups differ in the degree to which ERP correlates of recollection and familiarity support accurate memory decisions or is there evidence of compensatory recruitment of alternative neural activity in a-MCI? As prior studies in a-MCI have been confounded by group differences in overall performance, which could, for example, differentially dilute correct responses with “lucky guesses,” the current study was designed to better match recognition discrimination between the groups.
2. Are a-MCI patients less effective or efficient in retrieving familiarity- or recollection-based memories? This question more directly addresses the integrity of recollection and familiarity by comparing their putative ERP correlates for studied versus unstudied items, *regardless* of the accuracy of memory decisions for these items (“retrieval attempt” as opposed to “retrieval success”). To some extent, this comparison is akin to behavioral measures of familiarity and recollection in which a proportional estimate of success of these memory processes is calculated in the context of a given study and test condition. Most prior studies have simply examined retrieval success effects to make claims about the integrity of these processes, but we feel that such analyses speak more to the processes supporting successful memory rather than the effectiveness by which these memory traces are instantiated.
3. Do the neural correlates of familiarity and recollection based on study status correlate with behaviorally measured estimates of these processes? This question most directly tests the relationship between these ERP correlates and the memory processes they are thought to index. While a number of studies have supported the relationship between the early and late old/new effects with familiarity and recollection, respectively, there remains controversy in these mappings and, more generally, the dual process model (Squire et al., 2007; Voss et al., 2008, 2010a; Wixted et al., 2010). As cognitively normal (CN) older controls, who have been reported to have variable recollection, but spared familiarity relative to young adults (Parkin and Walter, 1992; Davidson and Glisky, 2002; Howard et al., 2006; Wolk et al., 2013), and patients with MCI likely represent a range of integrity for these memory processes (Wolk et al., 2008; Algarabel et al., 2009; Ally et al., 2009a; Hoppstadter et al., 2013), this is an ideal cohort for determining the relationship between behavioral estimates and the underlying neural substrates. In particular, a dissociation of these ERP components with their respective behavioral correlates would provide additional support for these mappings and, in turn, the general notion of the dual process model.

## MATERIALS AND METHODS

### SUBJECTS

Thirty-three CN older adults [mean age: 72.1 ± 8.9 (SD) years; mean education 16.8 ± 3.0 (SD) years] and 24 adults with a diagnosis of a-MCI [mean age: 70.0 ± 8.3 (SD) years; mean education 17.1 ± 2.8 (SD) years] participated in the study (one additional CN adult and two a-MCI patients were excluded due to poor quality ERP data). Subjects were recruited from the Alzheimer’s Disease Research Center (ADRC) of the University of Pittsburgh and the Alzheimer’s Disease Core Center (ADCC) of the University of Pennsylvania. As part of their enrollment in their respective centers, each patient underwent an extensive evaluation, including medical history and physical examination, neurological history and examination, and psychometric testing, usually including all elements of the National Alzheimer’s Coordinating Center’s (NACC) Uniform Data Set (Morris et al., 2006; Beekly et al., 2007; Weintraub et al., 2009). Clinical diagnosis was determined by review of the above data, in addition to relevant blood work and brain imaging, at a consensus conference attended by neurologists, neuropsychologists, and/or psychiatrists.

Diagnosis of a-MCI was made essentially following the criteria of Peterson and others (Petersen, 2004; Winblad et al., 2004). In addition to a subjective memory complaint, patients needed to have objective evidence of memory impairment for age. Strict cutoffs to denote impairment were not used, but generally patients performed greater than 1.5 SDs below age and education adjusted norms. Patients with a-MCI included those with isolated memory impairment (i.e., single-domain) and those with involvement of other aspects of cognition (i.e., multiple-domain). Consistent with the a-MCI designation, patients had to have minimal impairment in instrumental activities of daily living and not qualify for a diagnosis of dementia. Inclusion criteria were age between 50 and 85 years, >7 years of education, and English speaking from an early age. Participants were excluded if they had a history of clinical stroke, traumatic brain injury, alcohol, or drug abuse/dependence, prior electroconvulsive therapy, and any significant disease or medical/psychiatric condition that was felt to impact neuropsychological performance. The study was approved by the Institutional Review Boards of the University of Pennsylvania and the University of Pittsburgh.

For the purposes of this study, each subject completed the following psychometric battery within three months of the ERP recording: mini-mental status exam (MMSE; Folstein et al., 1975); digit span subtest of the Wechsler Adult Intelligence Scale III (Wechsler, 1987); category fluency (animals; Spreen and Strauss, 1998); Consortium to Establish a Registry for Alzheimer’s Disease (CERAD) word list memory (WLM) test (Morris et al., 1989); trail making test (TMT) A and B (Reitan, 1958); and a 15- or 30-item version of the Boston Naming Test (BNT; Kaplan et al., 1983).

### EXPERIMENTAL MATERIALS AND METHODS

We selected 480 nouns between five and eight letters (mean Kucera–Francis written frequency: 46.3) from the MRC Psycholinguistic database^1^. Eight study and test lists were created, counterbalanced by study status (half studied; half unstudied). Studied words were further counterbalanced by color (half red font; half green font) and number of study presentations [half once (1×); half thrice (3×)]. Words were presented on a computer screen in capital letters in red or green font during the study phase and white font in the test phase against a black background.

### EXPERIMENTAL PROCEDURE

#### Event-related potential paradigm

A source memory task was used to ensure that participants consciously attempted to recollect the prior study episode. The paradigm was divided into 20 study-test blocks. Each study block consisted of 14 words. The first and last two words served as “buffers” to reduce primacy and recency effects. Each word was preceded by a 1000 ms fixation (“+”). When the word was presented, subjects were instructed to decide if the referent was an object that was “animate” or “inanimate” to insure deep, semantic encoding. Subjects indicated their animate/inanimate choice by button press. The words “animate” and “inanimate” along with the button press mappings were displayed on the screen simultaneously with stimulus presentation. The study phase was self-paced with the word remaining on the screen until a response was made.

The test phase immediately followed study. For each block, 20 test items (10 studied; 10 unstudied) were presented. As with the study phase, each word followed presentation of a 1000 ms fixation (“+”). Subjects were asked to identify which words were “OLD” or “NEW” by button press with the mappings displayed under each word. Test items remained on the screen until a response was given. For words endorsed as “OLD,” a “GREEN or RED” prompt immediately followed in which subjects were instructed to recall the font color of each word and indicate with a corresponding button press. All aspects of the test phase were self-paced. For both the study and test phase, subjects were encouraged to respond as quickly as possible, but without sacrificing accuracy.

#### Behavioral paradigm

An additional behavioral paradigm was performed in a separate session to estimate recollection and familiarity. This task is a variant of the “process dissociation procedure (PDP)” and was previously described in prior reports involving some of the current cohort (Wolk et al., 2008, 2011). In brief, subjects studied words in either red or green font, analogous to the ERP paradigm. Test items consisted of previously studied words presented in white font and unstudied items. Participants were told to endorse only items studied in one of the two colors as “Old” (e.g., “Only endorse items that were previously studied in green as Old. Call all other items New.”). Using the language of the PDP, these are considered the “included” items. Words studied in the other font color, or the “excluded” items, produced a condition in which recollection opposes familiarity. As these words were previously studied, they may be associated with familiarity, potentially driving the subject to incorrectly endorse them as “Old.” However, the contextual retrieval of recollection would allow the subject to recall that the word had been studied in the other font color and correctly endorse it as “New.” Based on the rate of “Old” endorsements to these classes of items, one can calculate estimates of recollection (*R*) and familiarity (*F*) based on the following: *R* = *p*(included) – *p*(excluded); *F* = *p*(excluded)/(1 - *R*). To account for differences in base rates of false alarms (“Old” responses to novel words), familiarity was calculated using a measure of discrimination (*d*′) derived from signal detection theory (Yonelinas et al., 1995; Davidson et al., 2006). The delay between this task and the ERP paradigm was 44.2 ± 68.9 (SD) days.

### ELECTROPHYSIOLOGIC RECORDING

Subjects were fitted with an active two electrode cap (Behavioral Brain Sciences Center, Birmingham, UK). One hundred and twenty-eight Ag–AgCl BioSemi (Amsterdam, The Netherlands) “active” electrodes were connected to the cap in a pre-configured array placing each electrode in equidistant concentric circles from 10 to 20 system Cz position. In addition to the 128 scalp electrodes, electrodes were placed below and on the outer canthus of the left and right eye to measure vertical and horizontal electrooculography (EOG) activity. Electrical offsets were verified to be between *-*20 and 20 μV for every channel prior to data collection. Continuous electroencephalography (EEG) data were amplified and digitized with a sampling rate of 512 Hz and a default low-pass, anti-aliasing filter at one-fifth of the sampling rate^2^.

### EVENT-RELATED POTENTIAL PRE-PROCESSING AND STATISTICAL ANALYSIS

Data were processed off-line using the EMSE Software Suite (Source Signal Imaging, San Diego, CA, USA). A common average reference and 0.1–40 Hz bandwidth filter were applied. Trials were corrected for excessive EOG activity using the EMSE Ocular Artifact Correction Tool. After manual designation of artifact and artifact-free segments, a covariance technique models these segments, subtracting the contribution of the artifact from the recording when detected. Individual bad channels were corrected with the EMSE spatial interpolation filter, and trials with artifact exceeding approximately ± 90 μV were discarded.

**FIGURE 1:**
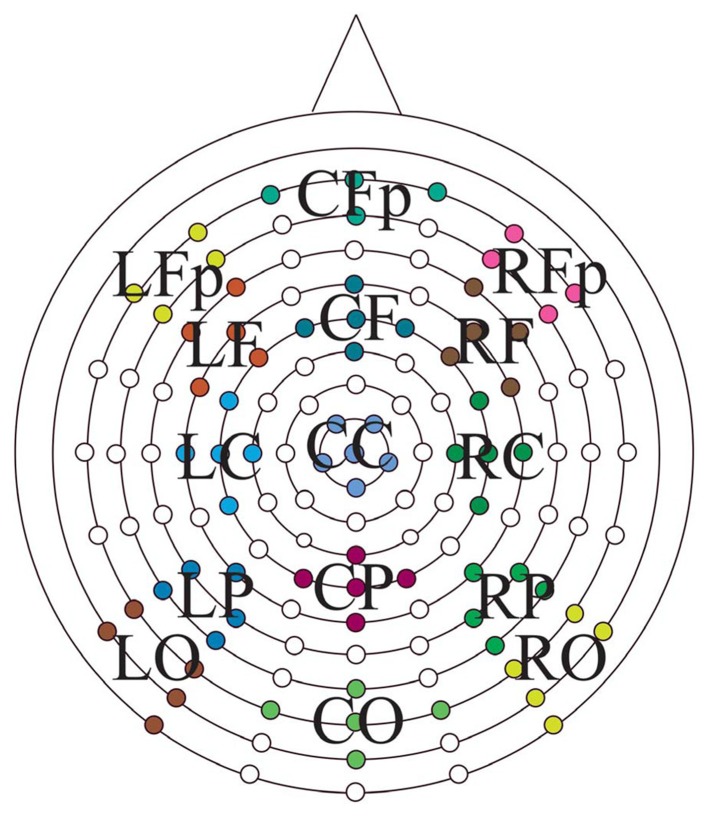
**Electrode positions on the Bio-Semi ActiveTwo headcap with the 15 regions of interest displayed**. Anterior-to-posterior: Fp, prefrontal; F, frontal; C, central; P, parietal; O, occipital central; left-to-right, L, left; C, midline; R, right.

Continuous EEG data were divided into epochs beginning 200 ms preceding test item presentation and ending 1500 ms after test item presentation. ERPs were calculated for the following stimulus classes to assess retrieval success effects: 1× hits, 3× hits, and correct rejections. In addition, ERPs of studied and unstudied items, regardless of response, were formed to determine group differences in the neural correlates associated with prior study. ERPs from individual electrodes were then averaged into 15 scalp locations divided into five anterior to posterior [prefrontal (Fp), frontal (F), central (I), parietal (P), occipital (O)] and three left to right (left, midline, and right) regions of interest (Figure 1). Mean peak amplitudes, relative to a 200 ms prestimulus baseline, were calculated for four epochs following stimulus presentation; 300–500, 600–800, 800–1200, and 1200–1500 ms. The first two intervals were chosen based on the established literature to differentially capture the early (FN400) and parietal (LPC) old/new effects. The 800–1200 ms interval was analyzed based on inspection of the data revealing a sustained parietal effect into this time frame, and the 1200–1500 ms interval was analyzed to address differences in the LFE.

Statistical analyses were performed in a standard manner using SPSS 20.0 (Chicago, IL, USA). In general, group differences were determined using analysis of variance (ANOVA). Selected electrode clusters for these analyses were determined based upon prior reports in the literature and visual inspection of the intervals of interest. The Greenhouse–Geisser correction procedure was used for repeated measures factors with greater than one numerator degree of freedom. Spearman correlation coefficients were calculated for determining the relationship between measures of recollection and familiarity determined in the separate behavioral task with their putative ERP correlates.

## RESULTS

### DEMOGRAPHIC AND PSYCHOMETRIC DATA

Demographic and psychometric data are presented in Table 1. The groups did not differ with regard to age or education. The overall cognitive impairment of the a-MCI group was relatively mild based on the MMSE (27.8), but significantly worse than that of the CN group (29.5) [*t*_(55)_ = 4.8; *p* < 0.01]. As a point of reference, the mean MMSE from the Alzheimer’s Disease Neuroimaging Initiative (ADNI) a-MCI cohort was 27.0 (Petersen et al., 2010). Consistent with their amnestic status, the a-MCI group displayed significant impairments in tests of memory relative to the CN participants. As anticipated in light of including both single and multiple domain patients, the a-MCI group was also significantly impaired on several non-memory tests. However, mean performance in these was generally within one standard deviation of the control group. Finally, the a-MCI group had a greater proportion of individuals who were carriers of the apolipoprotein E (ApoE) ε4 allele, the major genetic risk factor for AD (Mayeux et al., 1993), but this did not reach statistical significance (*p* > 0.1).

**Table 1:** Demographic and psychometric data.

	CN (*n* = 33)	MCI (*n* = 24)
Age (years)	72.1 (8.9)	70.0 (8.3)
Education (years)	16.8 (3.0)	17.1 (2.8)
Gender (% Female)	66.7	41.7
ApoE4 carrier status (%)	36.6	59.1
MMSE	29.5 (0.9)	27.8 (1.6)**
WLM immediate recall	24.0 (3.7)	16.8 (3.1)**
WLM delayed recall	8.2 (1.8)	2.6 (1.9)** WLM recognition	9.9 (0.3)	8.7 (1.4)**
Digit span forwards	7.0 (1.1)	6.5 (0.9)
Digit span backwards	5.4 (1.3)	4.6 (0.9)*
TMT A (s)	32.0 (12.2)	38.0 (14.8)
TMT B (s)	73.9 (28.7)	107.5 (54.8)**
Category fluency (animals)	22.0 (5.5)	16.6 (2.8)**
BNT	28.6 (2.2)	27.9 (2.7)

WLM immediate recall is the sum of the three immediate memory trials. WLM recognition is calculated as hits minus false alarms. Two CN did not complete TMT and digit span. BNT comparison includes 23 CN and 18 MCI patients, as the remainder performed a 15-item version of the task. ApoE genotype was not available in five individuals. *p < 0.05; **p < 0.01.

Behavioral performance on the memory task performed during EEG recording is displayed in Table 2. In both the 1× and 3× condition, the CN group displayed better discrimination and source memory. In an ANOVA of item memory discrimination with factors of study repetition (1×, 3×) and group, there was a main effect of repetition [*F*_(1,55)_ = 17.5, *p* < 0.001] and group [*F*_(1,55)_ = 41.2, *p* < 0.001], reflecting better discrimination in the 3× than 1× condition and for the CN group relative to a-MCI group. In addition, an interaction between repetition and group was observed [*F*_(1,55)_ = 8.7, *p* < 0.01] as the CN group appeared to benefit more from repetition than the a-MCI group; however, both groups displayed a significant repetition effect [CN: *t*_(32)_ = 13.1, *p* < 0.001; a-MCI: *t*_(23)_ = 9.3, *p* < 0.001]. A similar ANOVA was performed for source memory accuracy (proportion source correct/hits). Again, effects of repetition [*F*_(1,55)_ = 20.7, *p* < 0.001], group [*F*_(1,55)_ = 29.2, *p* < 0.001], and repetition × group [*F*_(1,55)_ = 18.3, *p* < 0.001] all reached significance, reflecting the better performance of the CN group and the benefit of repetition. Interestingly, the latter interaction was driven by the fact that only the CN group displayed improved source memory with study repetition [*t*_(32)_ = 6.3, *p* < 0.001] while the a-MCI group did not [*t*_(23)_ < 1.0].

**Table 2:** Performance on ERP and behavioral memory task.

	CN (*n* = 33)	MCI (*n* = 24)
Item memory (*d*’) 1×	2.55 (0.55)	1.60 (0.58)**
Item memory (*d*’) 3×	3.50 (0.71)	2.24 (0.83)**
Item memory (*d*’) Overall	2.88 (0.55)	1.88 (0.66)**
Source memory 1×	0.60 (0.10)	0.52 (0.08)*
Source memory 3×	0.70 (0.12)	0.53 (0.07)**
Source memory overall	0.66 (0.10)	0.53 (0.07)**
Recollection (proportion)	0.35 (0.23)	0.16 (0.17)*
Familiarity (*d*’)	1.52 (0.53)	0.77 (0.48)**

1×, test items with one prior study presentation; 3×, test items with three prior study episodes; *d*’ = discrimination; source memory is proportion source hits over total hits; estimates of recollection and familiarity from behavioral task in bottom two rows; *p < 0.01; **p < 0.001.

### RETRIEVAL SUCCESS EFFECTS

**FIGURE 2:**
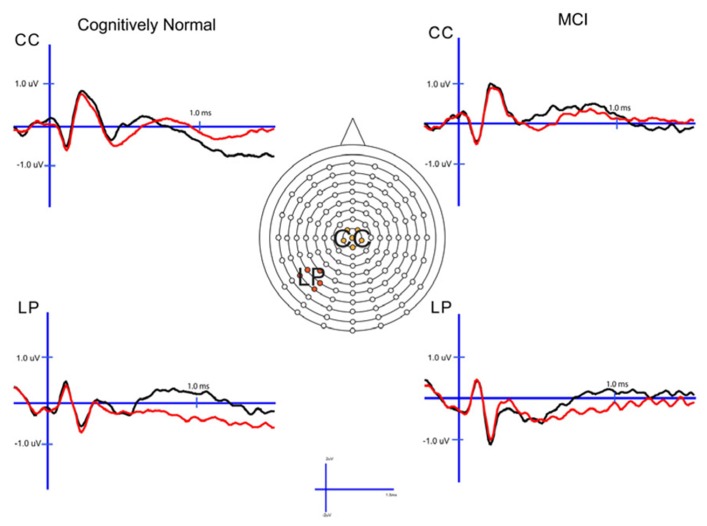
**Cognitively normal and amnestic mild cognitive impairment grand average ERP waveforms for hits (black) and correct rejections (red) in the midline central (CC) and left parietal (LP) sites**. Waves were generated from the 1× study condition in the CN group and the 3× study condition in the amnestic mild cognitive impairment group.

**FIGURE 3:**
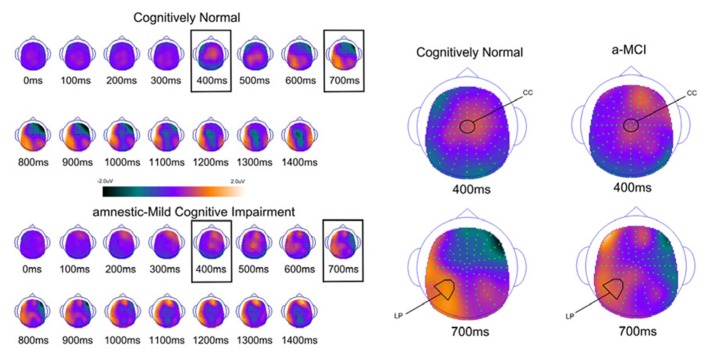
**Scalp topography maps for CN adults and patients with amnestic mild cognitive impairment for retrieval success effects (hits minus correct rejections) matched for performance**. Highlighted topographic maps are displayed on the right with electrode cluster used for analysis indicated. Each head map represents 100 ms average and color voltage scale is presented.

In order to examine the neural correlates of successful retrieval, we attempted to match the groups on item memory discrimination. We achieved reasonably comparable performance in the 3× condition for the a-MCI patients relative to the 1× condition for the CN group [*t*_(55)_ = 1.59, *p* > 0.1]. ERPs of hits and correct rejections and the scalp topographies of the difference waves for both groups in these respective conditions are displayed in Figures 2 and 3, respectively. As can be observed, both groups demonstrated a relative positivity associated with hits compared to correct rejections beginning at around 300 ms. From ~300 to 500 ms, this effect was maximal at central, midline scalp sites extending somewhat more anterior. Subsequently, there was a clear shift to a more posterior, left-hemisphere effect that slowly decreased throughout the remainder of the recording epoch. These hit/correct rejection differences have the timing and topography of the “early” (FN400) and “parietal” (LPC) old/new effects, with the exception of the early effect being less anterior than typically described. An additional right-hemisphere effect, most prominent in the CN group, emerges and moves more anterior near the end of the recording epoch consistent with descriptions of the LFE. Finally a central negativity is observed most prominently in the older adults in the later portion of the recording epoch, as has been described in other visual source memory paradigms as the late posterior negativity (LPN; Cycowicz and Friedman, 2003; Li et al., 2004).

#### Early (FN400) retrieval success effects

For comparison of the early old/new effect, a condition (hits, correct rejections) × group (MCI, CN) ANOVA was performed at the central electrode cluster (CC) that best captured this effect in the 300–500 ms epoch. There was a significant effect of condition [*F*_(1,55)_ = 13.3, *p* < 0.01] due to hits having a more positive voltage than correct rejections. There was no condition × group interaction [*F*_(1,55)_ < 1.0] suggesting the magnitude of the effect did not differ between the two groups although it was of somewhat decreased magnitude in the a-MCI patients (0.22 versus 0.38 μV).

#### Parietal (LPC) retrieval success effects

To assess the parietal old/new effect, the analogous ANOVA was performed on the left parietal cluster in the 600–800 ms interval. A significant effect of condition [*F*_(1,55)_ = 14.4, *p* < 0.001] reflected a more positive voltage for hits than correct rejections. Again, there was no interaction between condition and group, as the old/new effect was similar for both the CN and MCI groups (0.76 versus 0.58 μV, respectively). Given the apparent continuation of the left parietal effect beyond 800 ms, the same analysis was performed for the 800–1200 ms interval. Again, there was a significant effect of condition [*F*_(1,55)_ = 11.6, *p* < 0.01], but no interaction with group [*F*_(1,55)_ < 1.0]. Nonetheless, the magnitude of the effect was somewhat larger in the CN group (0.78 versus 0.51 μV).

#### Late posterior negativity and LFE retrieval success effects

Finally, given the apparent central negativity in the CN group consistent with the LPN, we also examined the old/new effect at the central cluster of electrodes for the 800–1200 ms interval. In this case, there was a main effect of condition [*F*_(1,55)_ = 4.8, *p* < 0.05] and a marginally significant interaction with group [*F*_(1,55)_ = 3.8, *p* < 0.06]. The interaction was driven by the presence of an LPN old/new effect in the CN group (-0.61 μV), but not a-MCI (-0.04 μV).

Based on inspection of scalp topography, it appeared that the a-MCI group may have exhibited a stronger and more central LFE than the CN group. To assess this potential difference, a condition (hits, correct rejections) × group (a-MCI, CN) × electrode cluster (LFp, CFp, RFp) ANOVA within the 1200–1500 ms interval was calculated. No main effect or interaction reached significance other than an effect of group [*F*_(1,55)_ = 6.1, *p* < 0.05] due to the a-MCI patients having generally more negative voltage in this time frame.

#### More rigorous matching of performance

Despite the lack of statistical difference between the groups based on item discrimination in the above analysis, it is worth pointing out that in absolute terms, the MCI group still performed more poorly. To address this possible confound, we removed the five highest performing CN participants and the three lowest MCI patients to achieve groups very closely matched in discrimination [*d*′: 2.40 versus 2.41 in CN versus MCI, respectively; *t*_(47)_ < 0.1]. There was no change in the results of any of the above analyses except related to the central negativity in the 800–1200 ms interval. With this subgroup, the old/new effect no longer reached a trend level group interaction [CN: -0.59 μV; MCI: -0.14 μV; *F*_(1,47)_ = 1.9, *p* > 0.1].

### RETRIEVAL ATTEMPT EFFECTS

**FIGURE 4:**
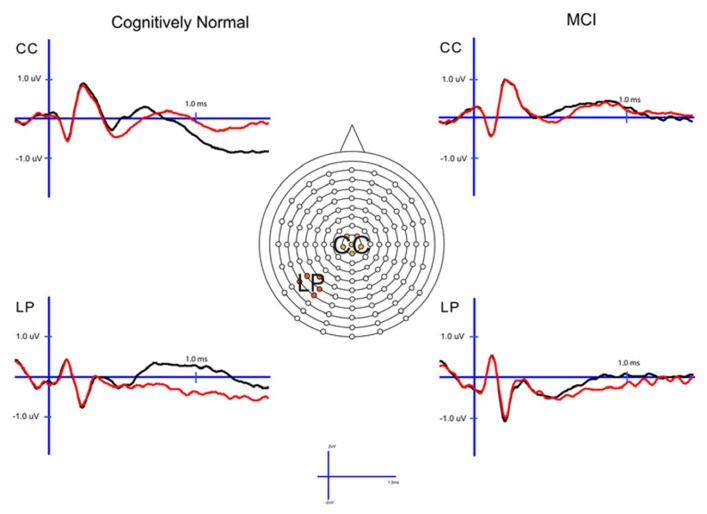
**Cognitively normal and amnestic mild cognitive impairment grand average ERP waveforms for studied (black) and unstudied (red) words in the midline central (CC) and left parietal (LP) sites**. Studied waves were generated by averaging across the 1× and 3× study condition.

**FIGURE 5:**
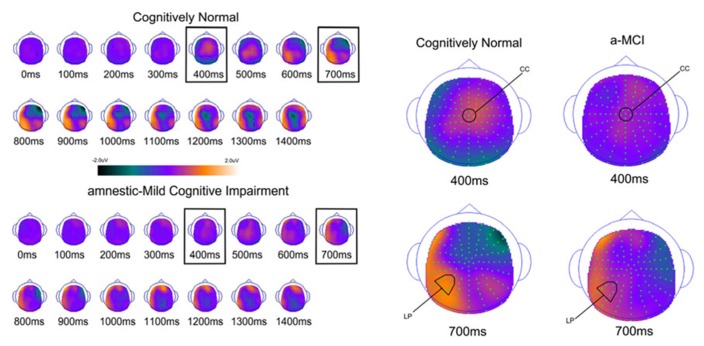
**Scalp topography maps for CN adults and patients with amnestic mild cognitive impairment for retrieval attempt effects (studied minus unstudied words) matched for performance**. Highlighted topographic maps are displayed on the right with electrode cluster used for analysis indicated. Each head map represents 100 ms average and color voltage scale is presented.

To determine how effective the groups are in retrieval of familiarity- and recollection-based memories, we compared all studied and unstudied items regardless of the accuracy of their associated memory judgments and not matched on performance. The ERPs of studied and unstudied items and the scalp topographies of the difference waves are displayed in Figures 4 and 5. As expected, the effect of condition was very similar to the above described differences when using only veridical responses. However, the early and late effects visually appeared somewhat diminished in the MCI group relative to CN participants.

#### Early (FN400) retrieval attempt effects

For comparison of the early old/new effect a study status (studied, unstudied) × group (a-MCI, CN) ANOVA was again performed at CC during the 300–500 ms epoch. There was a significant effect of study status [*F*_(1,55)_ = 15.6, *p* < 0.001] and a study status × group interaction [*F*_(1,55)_ = 6.0, *p* < 0.05]. These findings were driven by studied items being more positive than unstudied items, but the difference was larger in the CN group. To further evaluate this interaction, paired sampled *t*-tests revealed a significant studied/unstudied difference in the CN group [*t*_(33)_ = 4.7, *p* < 0.0001], but not in those with MCI [*t*_(23)_ = 1.0, *p* > 0.1].

#### Parietal (LPC) retrieval attempt effects

The analagous ANOVA at the left parietal cluster during the 600–800 ms interval revealed an effect of study status [*F*_(1,55)_ = 24.4, *p* < 0.0001] and a trend toward an interaction of study status and group [*F*_(1,55)_ = 3.7, *p* = 0.06]. Again, the effect of study status was driven by a more positive voltage for studied than unstudied items while the interaction appeared due to this effect being of greater magnitude in the CN group. Within group follow-up comparisons revealed that the LPC was highly significant in the CN group [*t*_(33)_ = 5.2, *p* < 0.0001], but of borderline significance in the a-MCI patients [*t*_(23)_ = 2.1, *p* = 0.05].

Analysis of the left parietal cluster during the 800–1200 ms interval also produced a significant main effect of study status [*F*_(1,55)_ = 18.8, *p* < 0.001] and an interaction of study status by group [*F*_(1,55)_ = 5.3, *p* < 0.05]. The interaction again appeared driven by a higher magnitude study status effect in the CN group [*t*_(33)_ = 5.5, *p* < 0.0001] than those with a-MCI [*t*_(33)_ = 1.4, *p* > 0.1].

#### Late posterior negativity and LFE retrieval attempt effects

As with the retrieval success analysis, we also examined the central cluster of electrodes in the 800–1200 ms interval to assess differences in the observed central negativity (i.e., LPN). There was both a significant effect of study status [*F*_(1,55)_ = 11.7, *p* < 0.01] and an interaction of study status with group [*F*_(1,55)_ = 9.2, *p* < 0.01]. This interaction was driven by a more negative response for studied than unstudied items in the CN group [*t*_(33)_ = 4.6, *p* < 0.001], which was not present in the a-MCI patients [*t*_(23)_ < 1.0].

Finally, we examined the LFE with a condition (studied, unstudied) × group (a-MCI, CN) × electrode cluster (LFp, CFp, RFp) ANOVA in the 1200–1500 ms epoch. While a main effect of condition was observed [*F*_(1,55)_ = 6.2, *p* < 0.05] due to more positive potentials associated with studied relative to unstudied items, there was no evidence of an interaction with group [*F*_(1,55)_ < 0.1].

### CORRELATIONS OF ERP COMPONENTS WITH RECOLLECTION AND FAMILIARITY

The FN400 and LPC effects have been argued to be associated with familiarity and recollection, respectively. Behavioral measures of these processes essentially measure their integrity (e.g., the proportion of items recollected) in the given context of the memory paradigm. Our retrieval attempt measures may provide the most analogous electrophysiologic correlate of this, as they index the degree to which these ERP components are instantiated over all test items, not just those with correct endorsements. Thus, we correlated retrieval attempt effects (i.e., studied minus unstudied voltages) with behavioral estimates of these memory processes derived from a very similar memory paradigm.

Behavioral recollection and familiarity estimates are displayed in Table 1 (bottom two rows). Consistent with our prior findings, patients with a-MCI displayed significantly lower recollection [*t*_(55)_ = 3.4, *p* < 0.01] and familiarity [*t*_(55)_ = 5.5, *p* < 0.001] measures than the CN group.

We found a significant correlation between the FN400 retrieval attempt effect at the central cluster of electrodes and the familiarity measure (*ρ* = 0.40, *p* < 0.01), but not with recollection (*ρ* = 0.20, *p* > 0.1). The opposite relationship was found for the LPC in the 600–800 ms epoch. No correlation was found with the familiarity measure (*ρ* = 0.07, *p* > 0.1), but a significant correlation was observed with recollection (*ρ* = 0.32, *p* < 0.05). This same relationship was also observed with the left parietal cluster in the 800–1200 ms interval (recollection: *ρ* = 0.44, *p* < 0.01; familiarity: *ρ* = 0.12, *p* > 0.1).

## DISCUSSION

The current findings address several important issues related to the nature of memory impairment and its electrophysiologic underpinnings in a-MCI, and they also clarify some of the inconsistencies in the literature. Most importantly, the current work revealed evidence of less reliable generation, or attenuation, of both the early (FN400) and late (LPC) ERP memory components in a-MCI patients. Prior work has suggested that these ERP effects are associated with familiarity and recollection, respectively, and the present findings are consistent with behavioral studies suggesting that both of these memory processes are impaired in a-MCI (Wolk et al., 2008; Algarabel et al., 2009; Ally et al., 2009a). The mapping of these components to their respective memory processes is further strengthened by the finding of an apparent double dissociation in the relationship between behavioral estimates of these processes obtained outside of the ERP recording session and the ERP retrieval attempt effects. Importantly, although a-MCI patients may be less effective in the retrieval of recollection and familiarity-based memories, as exhibited by the retrieval attempt analysis, the neural signature of successful memory did not differ significantly from CN adults. We will discuss each of these issues in turn.

### IMPLICATIONS FOR INTEGRITY OF RECOLLECTION AND FAMILIARITY IN a-MCI

A number of recent studies have examined the relative impairment of recollection and familiarity in a-MCI given its potential theoretical and clinical implications. Indeed, two of the earliest regions involved in the AD pathological process and associated with NFTs are the perirhinal and entorhinal cortices (Braak and Braak, 1991; Delacourte et al., 1999). These are structures that have also been argued to be essential for familiarity-based memory (Bowles et al., 2007; Yonelinas et al., 2007; Ranganath, 2010; Wolk and Dickerson, 2011; Wolk et al., 2011) and, thus, prodromal AD (i.e., AD in the a-MCI stage) may serve as a “lesion” model to test this hypothesis. Further, if this anatomic relationship is correct, impairment of familiarity could be an early feature of AD, qualitatively disparate from the relatively selective decline in recollection thought to be associated with “healthy” aging (Jacoby, 1999; Davidson and Glisky, 2002; Howard et al., 2006; although see Duarte et al., 2006). As such, familiarity might then be a relatively sensitive and specific marker for very early detection of AD-related pathology distinct from healthy age-associated memory loss (Wolk et al., 2013).

However, the literature has produced mixed results with regard to the integrity of familiarity in a-MCI patients using a variety of different process estimation approaches. Almost all of these studies report a significant impairment in recollection, or associative memory (Westerberg et al., 2006; Wolk et al., 2008; Ally et al., 2009a; Serra et al., 2010; Algarabel et al., 2012; Embree et al., 2012), consistent with the hippocampal involvement that is generally present in patients with a-MCI (Guillozet et al., 2003; Petersen et al., 2006). Alternatively, while several studies have reported impairment in familiarity (Wolk et al., 2008; Algarabel et al., 2009; Ally et al., 2009a; Embree et al., 2012), other work has suggested a relative sparing of this form of memory (Westerberg et al., 2006; Hudon et al., 2009; Serra et al., 2010). These conflicting data may be due to a number of factors, including patient characteristics, task difficulty, and assumptions inherent in the various methodologies for estimating familiarity. The latter issue is of particular relevance given controversies over the assumptions of each of these approaches. Thus, use of an “objective” electrophysiologic measure, not dependent on a defined stimulus or response class, is appealing.

An early (300–500 ms) frontocentral component, often referred to as the FN400, has been linked to familiarity-based memory. Our finding of a significantly reduced retrieval attempt effect (studied versus unstudied items) for this component in patients with a-MCI supports the notion that familiarity is impaired in this population. As noted above, the comparison of studied versus unstudied items, as opposed to hits versus correct rejections (i.e., retrieval success effect), is likely most analogous to a behavioral measure of the relative integrity of a particular memory process. In essence, this measure gives a metric of the average degree to which prior study influences these ERP components and an electrophysiologic metric of the integrity of the memory process indexed. Thus, while a-MCI patients may display a normal degree of familiarity when they correctly recognize a previously studied item as suggested by an intact early retrieval success effect, on average fewer items may engender this degree of familiarity than in CN adults.

There have been only a few prior studies that have assessed the integrity of this early component in a-MCI patients, and all but one have just examined retrieval success effects. Consistent with the current findings, Olichney et al. (2008) found a reduction in repetition effects within this epoch in a-MCI patients who later converted to clinical AD. In a study of retrieval success, a-MCI patients displayed an absent FN400 when words were used as stimuli, but not pictures (Ally et al., 2009b). The reduced early ERP effect for words is similar to the current findings and consonant with several behavioral studies that have found words particularly sensitive to failures of familiarity in a-MCI (Wolk et al., 2008; Ally et al., 2009a), but with relative sparing of familiarity for pictures (Westerberg et al., 2006; Embree et al., 2012), perhaps due to the enhanced perceptual and semantic encoding engendered by visually rich stimuli. Finally, a recent study reported absence of the FN400 in a-MCI and that this effect correlated with cortical MTL structures (Hoppstadter et al., 2013). In contrast, at least one prior study has suggested sparing of the FN400 effect even in mild AD, but, notably, this was a relatively small sample (*n* = 10) and only retrieval success effects were explored (Tendolkar et al., 1999). Furthermore, several studies of patients with clinical AD, as opposed to a-MCI, have revealed absence of both the FN400 and LPC (Friedman et al., 1992; Olichney et al., 2002), which may simply reflect the greater severity of underlying pathology in these patients.

Less controversial is the current finding of a diminished LPC effect in the a-MCI patients. This finding is consonant with the near universal finding of recollection impairment reported in behavioral studies of a-MCI (Westerberg et al., 2006; Anderson et al., 2008; Wolk et al., 2008; Ally et al., 2009a; Serra et al., 2010; Algarabel et al., 2012). Similarly, almost all ERP studies of this population have reported reduction in the LPC, thought to index this memory process (Olichney et al., 2008; Ally et al., 2009b; Hoppstadter et al., 2013; although see Schefter et al., 2012, for exception).

It is worth contrasting the finding of a diminished FN400 and LPC in a-MCI patients with reports of non-neurodegenerative amnesics who have isolated hippocampal lesions (Duzel et al., 2001; Addante et al., 2012). These studies have revealed sparing of the early component, but significant diminution of the LPC relative to controls. This set of findings can be argued to support the notion that the hippocampus is critical for recollection (indexed by LPC), but not familiarity (indexed by FN400), which has been posited to depend on perirhinal and entorhinal cortices. As the NFT pathology of early AD includes extrahippocampal MTL regions even prior to involvement of the hippocampus proper, the current finding is consistent with these mappings. Indeed, as noted above, a recent report has more directly related the FN400 to cortical MTL atrophy in an a-MCI cohort (Hoppstadter et al., 2013). It should also be pointed out that the CN group had a greater proportion of ApoE ε4 carriers than typical in the population and, thus, may be over-represented in individuals who could harbor preclinical AD. Given the early PRC involvement, this may have actually reduced the degree of FN400 difference with the a-MCI group observed, underestimating the degree of familiarity impairment.

These findings can also be placed within the context of recent work which has suggested that the anterior and posterior MTL appear to represent nodes of dissociable networks (Kahn et al., 2008; Didic et al., 2011; Libby et al., 2012; Ranganath and Ritchey, 2012). It has been argued that the anterior network, which includes the PRC, head of the hippocampus, ventral temporopolar cortex, and orbitofrontal cortex, may be linked to familiarity-based memory. Alternatively, the posterior network, which includes the body/tail of the hippocampus, parahippocampus, retrosplenial cortex, posterior cingulate, precuneus, and angular gyrus, is more tightly linked with the contextual and self-referential aspects of recollection (Ranganath and Ritchey, 2012). Scalp EEG recordings are unlikely to directly reflect activity from MTL structures and are more likely to be associated with cortical activity that is perhaps downstream of MTL processing. While the cortical localization based on scalp topography is dubious, it is tempting to note that that the anterior to posterior location of the FN400 and LPC is in keeping with the main cortical nodes of the anterior and posterior MTL networks and their mappings to familiarity and recollection, respectively.

As the anterior MTL is affected earliest with NFT pathology, derangements of the anterior network and its functions may represent the earliest features of AD and potentially an important biomarker at the preclinical and prodromal stages (Didic et al., 2011; Gour et al., 2011). Indeed, a number of studies have suggested that cognitive measures dependent on the integrity of the PRC and this anterior MTL network, including a series of studies assessing visual object recognition memory, may be sensitive to these early disease stages (Barbeau et al., 2004, 2008; Guedj et al., 2006; Didic et al., 2013; Wolk et al., 2013). Also a potential surrogate for this system, the FN400 may serve as a useful electrophysiologic biomarker.

### RETRIEVAL SUCCESS EFFECTS

Measurement of retrieval success effects (hits versus correct rejections) allows for assessment of the neural signature of veridical memory and is the approach most frequently examined in ERP studies of memory. By comparing a-MCI (enriched in patients with AD pathology) and CN adults, one can determine whether prodromal AD is associated with engagement of the same neural processes in support of accurate memory; however, as delineated above, this type of comparison does not necessarily reflect the effectiveness by which these processes or representations are instantiated across all test items. Nonetheless, one can assess whether alternative networks are recruited in support of successful memory with this type of analysis.

We found that for correct responses, patients with a-MCI displayed both an FN400 and LPC, which did not statistically differ from that of CN adults. This result suggests that similar neural generators support veridical memory in a-MCI patients as in CN adults despite the overall poorer memory of the former group. In other words, while a-MCI patients may be less effective in encoding and retrieving memories, when they do, similar neural representations are instantiated. In this case, additional study is required (3× condition) for the a-MCI patients to achieve similar performance and the concomitant neural signature of success as CN adults (1× condition).

As described above, most of the prior studies examining these effects in a-MCI have focused on retrieval success results with variable findings (Ally et al., 2009b; Schefter et al., 2012; Hoppstadter et al., 2013). One important difference between these prior studies and the current one is that we attempted to better match task performance. Retrieval success effects can be confounded by differences in performance due to “lucky guesses” contributing to both hits and correct rejections in the poorer performing group (Wilding and Rugg, 1996; Cansino et al., 2012; Wang et al., 2012). The attempt to more closely match performance in the current study, as well as the relatively high item discrimination, significantly mitigates against this concern.

The presence of an intact LPC effect may be considered somewhat surprising in the a-MCI patients given that their source memory was essentially at chance for items correctly identified as old. In light of the fact that the LPC is thought to index recollective, associative memory, one would expect that it would be related to accurate source memory decisions. One potential explanation for this apparent contradiction is that while a-MCI patients retrieved some associative details of the prior study episode, they did not tend to recollect the study color. In other words, they had non-criterial recollection relevant to the source memory task. Another possibility may be revealed by the lack of LPN in this group. While still of uncertain significance, this negative wave has been associated with visual source memory tasks and has been postulated to be related to recapitulation of study details at test, representing bound features of prior study (Cycowicz and Friedman, 2003; Johansson and Mecklinger, 2003; Li et al., 2004; Wolk et al., 2007). It is possible that in visual source memory tasks, this additional processing is necessary for accurate memory decisions. Finally, it is notable that from a quantitative perspective, the LPC effect was somewhat smaller than in the CN group, which may suggest that, in general, fewer associative details were actually retrieved. In light of the relatively low source accuracy of the CN group for this comparison (60%), a small difference in the LPC could result in the a-MCI group to approach chance performance. Further, as noted above, the somewhat higher than typical ApoE ε4 allele carrier rate in the CN group may have attenuated group differences.

Importantly, there was also no evidence of possible “compensatory” recruitment of alternative networks, including the LFE, which has been observed in CN older adults relative to young adults (Swick et al., 2006; Wolk et al., 2009). This result echoes that of Ally et al. (2009b) who also did not observe evidence of a compensatory effect when word stimuli were used, but did see such an effect with pictures.

### BEHAVIORAL–ERP CORRELATIONS

The current study used ERP indices of recollection and familiarity to estimate the integrity of these memory processes in a-MCI. While there is largely consensus in the literature that the LPC is related to recollection-based retrieval and even that its amplitude may be directly related to the amount of information recollected (Curran, 2000; Vilberg et al., 2006; Woodruff et al., 2006; Rugg and Curran, 2007; Wolk et al., 2007; Vilberg and Rugg, 2009), there is greater controversy surrounding the meaning of the FN400 (see Mecklinger et al., 2012; Paller et al., 2012 for opposing viewpoints). Indeed, Voss and colleagues have thoughtfully argued, based on several experiments, that this ERP index is more closely tied to conceptual priming and that the association with familiarity is driven, in large part, by the fact that these processes often co-occur (Voss and Paller, 2007, 2009; Voss et al., 2008, 2010b). In fact, the FN400 was named based on its very similar appearance to the N400, which has frequently been implicated in conceptual priming (Wolk et al., 2004). Adding to the complexity of this relationship is the notion that conceptual fluency may contribute to familiarity-based memory (Whittlesea and Leboe, 2000; Wolk et al., 2004) and that both conceptual priming and familiarity may be dependent on overlapping neural substrates (i.e., perirhinal cortex; Voss et al., 2009; Wang et al., 2010). Indeed, some may consider familiarity to be “non-episodic” in nature and to more closely align with semantic memory (Didic et al., 2011).

Further, it is also worth pointing out that our “retrieval attempt” analysis is essentially a repetition effect (studied versus unstudied items), as we did not conditionalize on the basis of response. As such, examination of these effects alone does not adjudicate between explicit or implicit correlates. However, the relationship between these ERP indices and behavioral estimates of recollection and familiarity, using a highly similar task performed outside of the electrophysiologic recording session, does provide support for their role in explicit memory. Namely, we found that the FN400 significantly correlated with a behavioral estimate of familiarity, but not recollection. Alternatively, the LPC correlated only with the behavioral estimate of recollection. This relative double dissociation supports the proposed mappings of these ERP components.

The only other report we know of that examined correlations between behavioral estimates of familiarity and ERP memory components was by Voss and Paller (2007). In this study, they attempted to control for effects of conceptual implicit memory by using “squiggle” stimuli rated for meaningfulness. They related estimates of familiarity, using the remember/know paradigm, to ERP old/new effects. Regardless of the level of meaningfulness of the stimuli, which were divided into a “high” and “low” group, they found no relationship with the FN400, but did show a significant correlation with a central and posterior effect in the 500–700 ms epoch, likely representing the LPC.

However, an important distinction between that analysis and the present one is that Voss and Paller related the familiarity measure to the hit/correct rejection difference (i.e., retrieval success). As argued above, behavioral estimates of familiarity measure the integrity of this memory process under particular test conditions while retrieval success is an index of the neural signature of successful memory. Thus, even if the FN400 does index familiarity, it is not clear that these measures will always correlate. For example, one could imagine a scenario in which, regardless of overall familiarity-based memory performance, whenever an item does engender an experience of familiarity that drives a correct response, a similar amplitude FN400 effect would be seen. If this were the case, this ERP index would not necessarily relate to the overall proportion of time that test items produced a feeling of familiarity. Nonetheless, it is worth pointing out that the correlation observed here between the FN400 retrieval attempt effect with the behavioral estimate of familiarity does not clearly disambiguate whether this ERP effect is related to conceptual priming, as it is possible that such priming effects are largest for items that engender a feeling of familiarity.

### CONCLUSION

The current data support the notion that despite similar neural mechanisms supporting successful memory, patients with a-MCI display impairment in both recollection and familiarity-based memory. The current findings are consistent with the topography of early AD pathology and the proposed anatomic substrates of these memory processes, as well as serving as electrophysiologic adjudication of conflicting behavioral results. As a-MCI is a heterogeneous condition in which only a subset of patients have prodromal AD, future work will need to determine the specificity of these findings for those that truly harbor underlying AD pathology. In particular, use of molecular biomarkers, such as imaging or cerebrospinal fluid measures for the presence of cerebral amyloidosis, would enhance interpretation of the current findings and should be pursued in future studies. Finally, the correlations of the FN400 and LPC with familiarity and recollection estimates, respectively, further support these mappings of the indices and the notion that they reflect dissociable memory processes.

## Conflict of Interest Statement

The authors declare that the research was conducted in the absence of any commercial or financial relationships that could be construed as a potential conflict of interest.

## Footnotes

1

2
